# Fluorescence-Lifetime Imaging Microscopy for Visualization of Quantum Dots’ Endocytic Pathway

**DOI:** 10.3390/ijms17040473

**Published:** 2016-03-30

**Authors:** Leona Damalakiene, Vitalijus Karabanovas, Saulius Bagdonas, Ricardas Rotomskis

**Affiliations:** 1 Biophotonics Group of Laser Research Center, Faculty of Physics, Vilnius University, Sauletekio 9, b. 3, Vilnius LT-10222, Lithuania; leona.bandzaityte@gmail.com (L.D.); saulius.bagdonas@ff.vu.lt (S.B.); ricardas.rotomskis@ff.vu.lt (R.R.); 2 Biomedical Physics Laboratory, National Cancer Institute, P. Baublio 3b, Vilnius LT-08406, Lithuania; 3 Department of Chemistry and Bioengineering, Vilnius Gediminas Technical University, Vilnius LT-10223, Lithuania

**Keywords:** quantum dots, fluorescence-lifetime imaging, endocytosis, intracellular distribution

## Abstract

Accumulation of carboxylated polyethylene glycol (PEG) CdSe/ZnSquantum dots (QDs) has been monitored in living fibroblasts using confocal microscopy for fluorescence intensity and fluorescence-lifetime imaging (FLIM). The wide range of mean photoluminescence (PL) lifetime values was observed for the intracellular QDs in different intracellular microenvironment, which revealed structural heterogeneity of endosomes and enabled the distinguishing among endosomes of different maturity.

## 1. Introduction

Fluorescence microscopy is a powerful technique widely used for *in vitro* and *in vivo* research of biological processes in real time at the cellular and subcellular levels. It is highly sensitive to the changes at the molecular level and ensures high spatial resolution of the collected data. However, the analysis of the fluorescence results gained from a biological object is often complicated due to such limiting factors as light scattering and absorption. Fluorescence-lifetime imaging (FLIM) has emerged as one of key techniques allowing visualizing the interaction of nanoparticles and other fluorophores with surrounding environment in living cells [1,2,3]. The fluorescence lifetime provides an absolute measurement, which, compared to fluorescence intensity, is less susceptible to artefacts arising from scattered light, photobleaching, non-uniform illumination of the sample, light path length, or intensity variations of excitation [2]. Fluorescence-lifetime imaging microscopy (FLIM), the benefits of which are already well summarized [4,5], is a powerful tool with a possibility of high resolution and multiplexing [6] for the determination of the spatial location of nanoparticles and the examination of their microenvironment and interactions [7,8]. The prospective combination of different nanoparticles and FLIM techniques had already been demonstrated for the *in vitro* and *in vivo* sensing and imaging. The twin application of nanoparticles and FLIM enables a functional insight into cellular processes, which could be directly visualized and analysed in the living cell. Recent examples include a nanoscaled fluorescent polymeric thermometer for intracellular temperature mapping [9], dye-loaded polystyrene nanoparticles for photoluminescence lifetime multiplexing and bar-coding of cells in co-cultures [10], monitoring of *in situ* release of drug doxorubicin from nanoparticles [11], *in vivo* imaging with near-infrared emitting [12], and nanoparticles and diamond nanoparticles [13].

In particular, quantum dots (QDs), due to long photoluminescence (PL) lifetimes and multi-exponential decay patterns [14], have a great potential for multiplexing and time-gated detection of biologically important intracellular targets with enhanced selectivity and sensitivity. Recent studies suggest that mercaptopropionic acid (MPA)capped CdSe/ZnS QDs [15] and carboxylated QDs [16] could be promising pH nanosensors being applied as intracellular probes to examine photoluminescence kinetics with the aid of FLIM technology for the imaging of different labelled areas, bearing distinct lifetimes inside living cells. Moreover, QDs are exploited as donors in resonance energy transfer to conventional photosensitizers used in the photodynamic therapy of cancer, and it was demonstrated that FLIM provides additional information on intracellular localization of the QD-chlorin e6 complex [17,18].

In this work, we demonstrate that the application of fluorescence-lifetime microscopy to study the time-dependent accumulation dynamics of carboxylated QDs in living cells enables the observation of the sub-structures present in endosomes at different stages of their maturity.

## 2. Results

**Figure 1:**
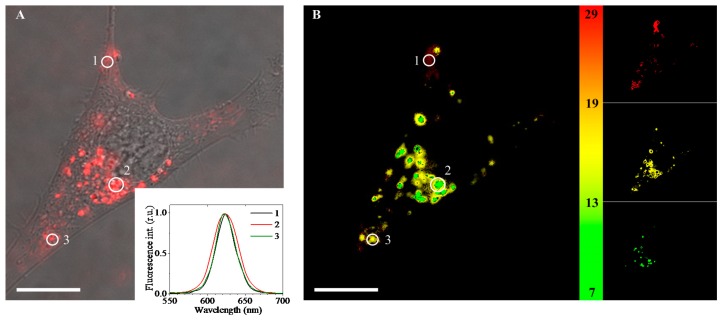
Microscopy images of immortalized mouse embryonic fibroblast (NIH3T3) cells incubated with quantum dots (QDs) for 3 h: (**A**) overlaid phase contrast and confocal fluorescence images, an inset—normalized fluorescence spectra of the cell’s areas marked with 1, 2 and 3 white circles; and (**B**) overlaid fluorescence-lifetime images in different lifetime decay gates (7–13 ns—green, 13–19 ns—yellow and 19–29 ns—red); right panel shows fluorescence-lifetime imaging (FLIM) images in green, yellow and red gates separately. Scale bar: 5 μm.

Confocal fluorescence microscopy pictures showed usual vesicular distribution [19] of endocytosed red-luminescing QDs (the peak intensity is at 623 nm) in the cell cytoplasm after 3 h of incubation (Figure 1A).

However, FLIM images taken at three defined time gates enabled the registering of the spatial heterogeneity of the intracellular vesicles (Figure 1B and the insets). The presence of time-related differences in the PL lifetime intracellular distribution implies that the intravesicular QDs are surrounded with different microenvironments [19]. Otherwise, if the QDs were in the homogeneous environment, the FLIM images would be identical, and only the intensity would vary.

**Figure 2:**
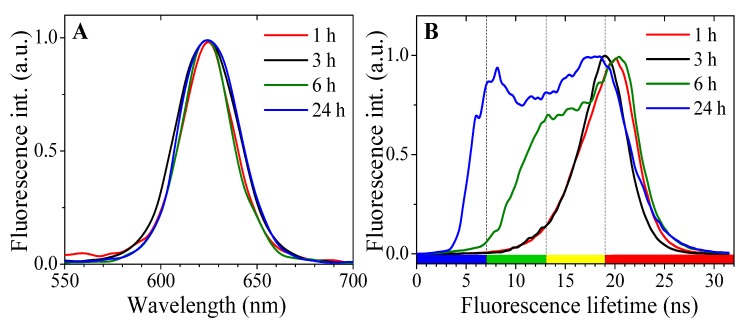
(**A**) Normalized fluorescence spectra of the intracellular vesicles after different QDs incubation times; and (**B**) normalized distribution of mean photoluminescence (PL) lifetimes of QDs localized inside NIH3T3 cells at different time intervals.

Our previous studies have demonstrated the time-dependent endosomal accumulation of QDs [19] and related gradual changes in morphology of endosomes. Moreover, our earlier studies employing endocytotic markers [20] confirmed that CdSe/ZnS carboxylic-coated QD enter fibroblast cells via lipid raft/caveolin-mediated endocytosis, pass early sorting endosomes, and accumulate in the multivesicular bodies. Therefore, investigating whether the different phases in uptake and trafficking of QDs could be identified with a FLIM method as well was of interest. The changes were not observed in photoliuminescence spectra (Figure 2A), but indeed detected in the distribution of mean PL lifetimes of intracellular QDs after different incubation times (Figure 2B).

After 1 to 3 h of incubation (when QDs are either adherent to the membrane, or are in the small—up to 1 μm in diameter—endocytic vesicles distributed in cytoplasm [19]), the PL lifetimes are mainly in the range of 16–22 ns. After longer (<6 h) incubation, when larger endosomes (~2–3 μm in diameter) with QD are concentrated in a perinuclear region [19], an additional range of shorter PL lifetimes (10–15 ns) was registered. Additionally, for the cells in the saturation stage—after incubation for 24 h, when the multi-vesicular body-like structures (~3–8 μm) are formed and distributed in the cytoplasm [19]—the increased input of even shorter (5–9 ns) PL lifetimes was detected (Figure 2B). Thus, Figure 2 clearly shows that the mean PL lifetimes of the intracellular carboxylated CdSe/ZnS QDs greatly vary depending on the changes of intracellular microenvironment, which are related to both the QDs incubation time and their location within the cell. The QDs, detected in the vicinity to the cell plasma membrane, had longer mean lifetimes compared to those being accumulated in endosomes.

**Figure 3:**
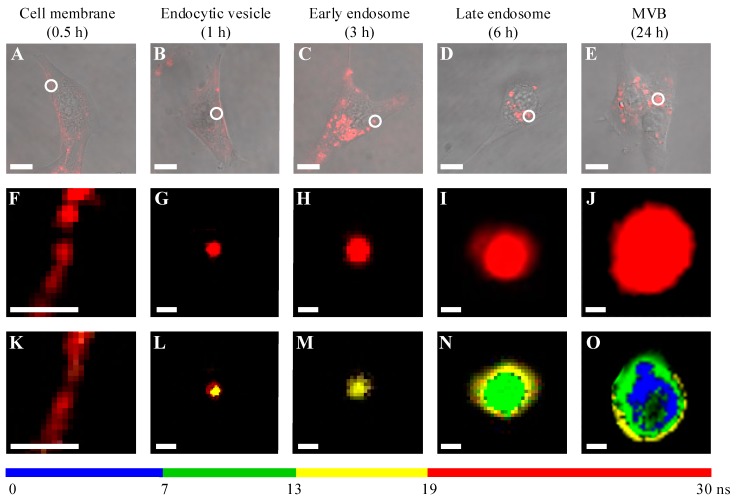
Overlaid phase contrast and confocal photoliuminescence images of NIH3T3 cells at different times of incubation with QDs revealing their intracellular distribution: (**A**) QDs adherent to the membrane; (**B**,**C**) endocytotic vesicles and early endosomes distributed in the cytoplasm; (**D**) late endosomes are concentrated in a perinuclear region; (**E**) multi-vesicular body-like structures distributed in the cytoplasm; scale bar: 10 μm. Confocal fluorescence images and fluorescence-lifetime images of areas and vesicles marked with white circles in (**A**–**E**): membrane (**F**,**K**); endocytotic vesicle (**G**,**L**); early endosome (**H**,**M**); late endosome (**I**,**N**); and multi-vesicular body (MVB) (**J**,**O**); scale bar: 1 μm.

The differences in fluorescence lifetimes were employed to monitor the intracellular trafficking of QDs in detail through all previously demonstrated stages of intracellular accumulation [19,20], starting from the plasma membrane to the early endosomes (<3 h), late endosomes (~6 h), and multi-vesicular bodies (MVBs, ~24 h) (Figure 3). It is notable that the PL spectra of QDs being localized inside the vesicles at different places (Figure 1A, inset) or those at any level of maturity (Figure 2A) were found to be almost the same. However, FLIM enabled the distinguishing of QDs accumulated among different endosomes. At the first phase, when QDs adhere to the plasma membrane (Figure 3A,F), the QDs mean PL lifetimes are the longest (Figure 2B and Figure 3K). When QDs are internalized into immortalized mouse embryonic fibroblast (NIH3T3) cells, and the endocytic vesicles fully-filled with QDs are formed, the shorter mean PL lifetimes appear in the inner volume of the vesicle, which is still surrounded by the external structure exhibiting the longest PL lifetimes like those of QDs interacted with plasma membrane (Figure 3B,G,L). An early endosome containing QDs had a quite homogenous structure (Figure 3C,H,M), possessing only the shorter lifetimes. The intracellular vesicles bearing more complex structures appeared after the fusion of early endosomes with late endosomes (Figure 3D,I,N). FLIM images revealed random distribution of different lifetimes in the outer area and even shorter lifetimes in the inner volume of the vesicles. The heterogeneous structure of multi-vesicular bodies was uncovered in the image when three time gates were applied, and the shortest average lifetimes were detected in the central area (Figure 3E,J,O—shown in blue). The green area seen in the middle most likely corresponds to a mixture of invaginated membranes and small intraluminous vesicles, as the accumulation of internal membranes in vesicles starts at the stage of an early endosome and is thought to continue as it “matures” to a late endosome [21].

## 3. Discussion

**Figure 4:**
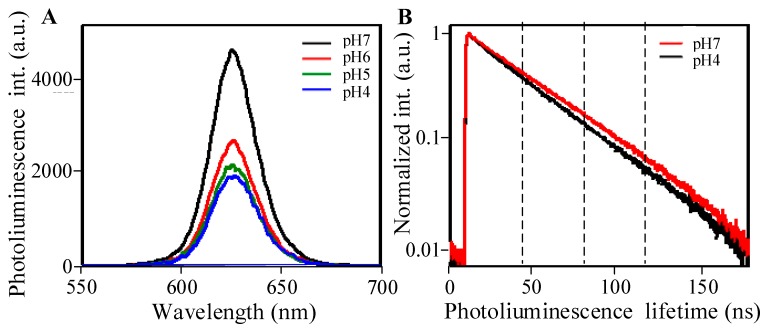
(**A**) Photoliuminescence spectra of the QDs water suspension of different pH values; and (**B**) PL lifetimes of QDs at different pH.

One important environmental factor affecting PL lifetime of intracellular QDs is the medium pH. By studying pH dependence of the PL of QDs in living cells, it was found that cellular environment of low pH quenches QD photoluminescence, [16] and that mean PL lifetimes being measured in membrane, cytoplasm, or endosomes were shorter in more acidic pH [15]. Our measurements of QDs’ PL lifetime, which were performed in water suspensions of different pHs, also demonstrated that reduction in pH value definitely affects the QDs’ fluorescence intensity (Figure 4A) and shortens PL lifetime (Figure 4B). However, the shortest values in acid solutions did not fall below 14 ns (Table 1), whereas during maturation of endosomes QDs’ PL lifetime reached ~7 ns.

Furthermore, other studies have noted that the lifetime of QDs in the acidic lysosomes (pH 4.5–5.0) was even shorter than that in a more acidic aqueous solution with a pH value of 2 [22]. Thus, the time-dependent shortening of PL lifetime τ in the case of intracellular QDs in endosomes cannot be attributed only to the changes in local pH, implying the particular transition of their internal environment.

The photophysical properties of semiconductor QDs are sensitive to the processes taking place on their surface [7]. It is well-known that, in general cases, the PL lifetime *τ* is inversely proportional to the transition rate *k* = *k_r_* + *k_nr_*, where *k_r_* and *k_nr_* are the rates of radiative and non-radiative decay, respectively. А higher non-radiative decay rate will result in reduction of PL quantum efficiency and a shorter PL lifetime [23]. The interaction between external biomolecules and those of QDs coating may also enhance non-radiative decay and lead to the shortening of the mean PL lifetimes. The analysis of PL decays of QDs in phosphate buffer saline (PBS) (pH 7.4) containing different amino acids or proteins indicated that the higher concentration of amino acids resulted in a shorter PL lifetime [23]. The lumen of early recycling endosomes has a pH value of 6.5–6.4 (as compared to pH 7.2 in the cytosol), that of late multi-vesicular endosomes has a pH of 6.0–5.0, and, after fusing with lysosomes, a pH of 5.0–4.5 is reached [22]. Moreover, the reduction of PL lifetimes was also observed for negatively charged QDs, which interact with positively charged intracellular proteins [24]. The difference between lipid composition of late endosomes and that of earlier endocytic compartments has been reported, the former being enriched in triglycerides, cholesterol esters, and selected phospholipids [21]. Compared to the limiting membrane, the internal membranes of late endosomes/MVBs was found to be heavily enriched in highly hydrophobic, cone-shaped phospholipids dedicated to bend membranes for formation of MVB [21]. One more assumption concerning the appearance of the shortest lifetimes detected at the last stage of QDs accumulation can be made on the grounds that QDs are more tightly packed in the mature endosomes and therefore can undergo a reduction in lifetime as a result of the energy transfer between QDs of different sizes in the ensemble of nanoparticles [25].

Since the outer stabilizing layer (coating) of studied QDs is made of polyethylene glycol (PEG) with ionizable carboxylic acid groups, the interaction capacity of QDs also becomes pH-sensitive. Under low-pH conditions, the fraction of protonated COOH groups increases, which facilitates the formation of hydrogen bonds between the QDs coating and the biomolecules, [26] also facilitating closer interaction between QDs.

Thus, the changes in mean PL lifetimes of QD observed during their accumulation most likely are caused by the variations in biomolecular composition and acidity of the intracellular microenvironment altering molecular interactions of the QDs coating, which can affect the intrinsic photoluminescence characteristics of nanoparticles.

## 4. Materials and Methods

The non-targeted quantum dots eFluor™ 625 NC were purchased from eBiosciences, San Diego, CA, USA. These QDs with a photoluminescence (PL) peak at 625 nm have a CdSe core passivated with a ZnS shell and are capped with a micelle-like coating of amphiphilic 1,2-distearoyl-sn-glycero-3-phosphoethanolamine-*N*-[carboxy(poly-(ethylene glycol))-2000], *i.e.*, DSPE-PEG2000 (Avanti Lipid, Alabaster, AL, USA, functionalized with carboxyl groups resulting in ~20 nm hydrodynamic radius [20]. The 10 nM concentration of QDs was used for the incubation of cells in accumulation experiments. The accumulation and distribution of the non-targeted quantum dots eFluor™ 625 NC in the cells as well as investigation of the molecular mechanisms is presented in [19]. The culturing of immortalized mouse embryonic fibroblast—NIH3T3—cells and the application of confocal microscopy for live cell imaging were performed as described previously [19]. 

Fluorescence-lifetime images were acquired using Lifetime and FCS Upgrade for Nikon C1si (PicoQuant GmbH, Berlin, Germany). The system consisted of a 405-nm pulsed diode laser with a pulse width of 39 ps and a repetition rate of 10 MHz, which allowed for the use of a measurement time interval of 100 ns. Detected photons were counted by a time correlated single-photon counter PicoHarp 300 (PicoQuant GmbH). Initialization, scanning, and data acquisition were controlled by the same Nikon C1si microscope system. PL lifetime signal of QDs in living cells and in water suspensions of the different pH values was detected with a single channel unit of single photon-counting avalanche photodiodes (SPADs) at a spectral range of 578 ± 52.5 nm. Each lifetime image was obtained by collecting 1000 counts at the peak value, and the image resolution was fixed at 512 × 512 pixels. FLIM images were reconstructed using a three-exponential fitting model (SymPhoTime software, PicoQuant GmbH), in which an average lifetime of QDs is displayed for every given pixel. Histograms for all average lifetimes (for all pixels) of QDs were plotted using the SymPhoTime v.5.2 (PicoQuant GmbH) software.

## 5. Conclusions

The fluorescence spectra as well as confocal fluorescence microscopy images of intracellular distribution of carboxylated CdSe/ZnS-PEG QDs were found to be very similar despite the fact that during observation period QDs were translocated to different organelles. It was demonstrated for the first time that FLIM enabled the distinguishing of QDs accumulated among different endosomes. At the first phase of accumulation, when QDs adhered to the plasma membrane, the PL lifetimes were the longest. When QDs are internalized into endocytic vesicles, the shorter lifetimes appeared. The vesicles bearing more complex internal structures with several mean PL lifetimes appeared at the last phase of accumulation.

Two of the possible reasons for the shortening of PL lifetimes of intravesicular QDs are the acidic medium and the altered interactions of QDs with surrounding biomolecules in the heterogeneous microenvironment.

The observed spatial heterogeneity of organelles implies the possibility of applying QDs in combination with FLIM technique for the imaging of the sub-structure of endosomes and the accurate distinction between early and late endosomes on the basis of morphology, when appropriate time gates are chosen, but much more effort is needed to relate the PL lifetimes of intracellular QDs with particular biomolecular structures.

## Figures and Tables

**Table 1:** Mean photoluminescence (PL) lifetimes of quantum dots in water suspensions of different pH values.

pH Value	pH 4.0	pH 4.5	pH 5.0	pH 5.5	pH 6.0	pH 7.2
PL lifetime τ (ns)	14.7 ± 0.6	15.7 ± 0.5	15.7 ± 0.5	16.5 ± 0.50	17.6 ± 0.2	19.0 ± 0.6

## Abbreviations

QDs	Quantum dots
PL	Photoluminescence
FLIM	Fluoscence-lifetime imaging microscopy
PBS	phosphate buffer saline
NIH3T3	immortalized mouse embryonic fibroblast cells
PEG	polyethylene glycol
MPA	mercaptopropionic acid
